# Seed priming-induced enhancement in seed germination, Seedling vigor, and productivity of foxtail millet (*Setaria italica* L.) in winter and summer seasons under Bangladesh conditions

**DOI:** 10.1371/journal.pone.0348288

**Published:** 2026-04-29

**Authors:** A. K. M. Mominul Islam, Tamanna Khatun, Prianka Chanda Bipra, Md. Sazzad, Tapon Kumar Roy, Md. Masud Rana, Sabina Yeasmin, Sinthia Afsana Kheya, Sanjida Afrin Urmi, Mst. Masuma Momtaj Meem, Md. Parvez Anwar, A. K. M. Aminul Islam

**Affiliations:** 1 Department of Agronomy, Bangladesh Agricultural University, Mymensingh, Bangladesh; 2 Entomology Division, Bangladesh Rice Research Institute, Gazipur, Bangladesh; 3 Department of Entomology, Bangladesh Agricultural University, Mymensingh, Bangladesh; 4 Department of Genetics and Plant Breeding, Gazipur Agricultural University, Gazipur, Bangladesh; University of Agriculture Faisalabad, PAKISTAN

## Abstract

Foxtail millet is a nutritionally rich and climate-resilient cereal crop; however, poor germination and weak early seedling growth often limit its productivity. This study evaluated the effects of seed priming on germination, seedling vigor, yield attributes, and grain yield of foxtail millet through laboratory and field experiments. In the laboratory study, four foxtail millet varieties were subjected to six priming chemicals at two concentrations each, along with hydropriming and an unprimed control. Seed priming significantly influenced all germination and seedling vigor traits. The highest germination percentage (86.44%) and germination index (116.49), were recorded under NaCl priming at 10000 ppm. Maximum seedling vigor index (6.08), speed of emergence (86.51), germination energy (61.63) were achieved with NaOCl at 500 ppm, while the lowest germination performance occurred under CaCl_2_ at 20000 ppm. The shortest time to 50% germination (T_50_ = 1.55 days), mean germination time (MGT = 4.54 days) and seedling vigor index were obtained from no priming, while the longest T_50_ (2.0 days) and MGT (4.78 days) were recorded with KNO_3_ at 30000 ppm. The longest shoot (3.76 cm) and shoot dry weight (28.96 mg) were obtained with KNO_3_ at 15000 ppm, while the longest root (3.92 cm) and seedling length (7.51 cm) were recorded under NaOCl at 1000 ppm. The lowest shoot length (2.18 cm), root length (2.08 cm), seedling length (4.26 cm), shoot (12.03 mg), root (10.66 mg) and seedling (22.69 mg) dry weight were obtained from no priming. Based on laboratory performance, selected treatments were evaluated under field conditions. During the winter season, the highest grain (2.72 t ha^-1^) and straw (5.03 t ha^-1^) were recorded from BARI Kaon-2 × NaCl (10000 ppm). The highest grain yield obtained under this treatment combination was due to the production of the highest values for ear length (17.83 cm), ear weight (14.30 g), filled grains ear^-1^ (2918.33) and 1000-grain weight (2.56 g). Whereas, the lowest grain yield (1.17 t ha^-1^) was given by BARI Kaon-1 × no priming. In the summer season, the highest grain yield (3.93 t ha^-1^) was obtained from BARI Kaon-1 × NaCl (10000 ppm) this was due to the production of the higher values for most of the yield attributes by this treatment combination. In summer season, the lowest grain yield was obtained from BARI Kaon-4 × no priming. In conclusion, BARI Kaon-2 × NaCl (10000 ppm) performed best during the winter season, whereas BARI Kaon-1 × NaCl (10000 ppm) exhibited superior performance during the summer season. Seed priming with NaCl (10000 ppm) emerged as a seasonally robust strategy to improve germination, crop establishment, and yield of foxtail millet in Bangladesh.

## 1. Introduction

Foxtail millet (*Setaria italica* L.) is an important small millet crop known for its ability to thrive in various ecological conditions, its low input needs, and its resilience to stresses like drought and high temperatures [1,2]. This crop is a nutritionally rich, gluten-free cereal valued for its high protein, dietary fiber, and essential minerals such as iron, calcium, and potassium. It also has notable antioxidant properties, making it a key food source to help fight malnutrition in Bangladesh and other areas facing similar dietary issues [3].

Millets are mainly grown in northern, north-western, central, and hilly regions of Bangladesh, with 9.3 thousand hectares cultivated in the fiscal year 2020–2021, resulting in a total production of 9.6 thousand metric tons [4]. However, in the last decade, the area and production of millets have dropped significantly, decreasing by 73% and 19%, respectively since 2010 [4]. Despite its potential to aid in climate-resilient agriculture, diversify cereal-based cropping systems, and foster sustainable farming on marginal lands, foxtail millet remains underutilized in Bangladesh [4].

A major challenge in growing foxtail millet is poor seed quality, which often leads to low and uneven germination, weak plant establishment, and reduced crop density [5,6]. These issues become more apparent during the winter and summer seasons in Bangladesh, where varying temperatures, moisture levels, and soil conditions negatively impact early crop growth [7]. Since strong seedling growth is crucial for final yield, improving germination and early growth is essential for boosting millet productivity [8].

Seed priming is a treatment applied before sowing that involves controlled watering to start the physiological and biochemical processes needed for germination while inhibiting radicle emergence [9]. This method has been shown to boost germination rates, shorten germination time, and enhance seedling vigor in cereals and millets. Different priming methods, such as hydropriming, osmopriming, and chemical priming, have shown beneficial effects on enzyme activity, membrane repair, and metabolic efficiency, which lead to better early growth and stress tolerance [10]. However, the effectiveness of seed priming is greatly affected by the crop’s genotype, the type of priming agent used, and the environmental conditions, underscoring the need for region-specific studies. In Bangladesh, research on seed priming for foxtail millet is limited, especially regarding the comparison of various priming agents in laboratory settings and their validation in field conditions [4]. Furthermore, seasonal differences between winter and summer periods may affect how well priming treatments work because of variations in temperature, humidity, and soil moisture. Thus, it is crucial to identify a potential priming agent that reliably enhances seed performance across different seasons for practical field applications.

This study aimed to fill these knowledge gaps using a systematic approach. First, laboratory experiments were conducted to examine the effects of various seed priming agents on germination traits and seedling vigor in foxtail millet. The field experiments sought to evaluate the impact of selected seed priming agent on growth, yield components, and grain yield in the specific agro-climatic conditions of Bangladesh. The findings from this research are expected to offer practical insights into seed priming methods for foxtail millet and support the creation of low-cost, farmer-friendly techniques to enhance crop establishment, growth, and yield.

## 2. Materials and methods

### 2.1. Experimental site and duration

The laboratory experiment was conducted at the Agro-Innovation Laboratory (24°43’24.0852“, 90°26’11.8572”), while the two field experiments were conducted at the Agronomy Field Laboratory of Bangladesh Agricultural University (BAU), Mymensingh, during the winter (24°43’11.103”, 90°25’36.6168”) and summer (24°43’11.91”, 90°25’43.9818”) season (October 2024 – May 2025). The experimental field is located in the Old Brahmaputra Floodplain (AEZ-9) of Bangladesh which is characterized by fertile alluvial soils suitable for millet cultivation.

Both the field experiments were conducted in adjacent fields rather than the same field. The field belongs to non-calcareous dark-grey floodplain soil. The land was medium high land and the soil was silty-loam and moderately fertile. The analytical soil properties of both experimental fields have been presented in Table 1, which show no significant differences. The climate of the locality was tropical in nature characterized by high temperature, high humidity and heavy precipitation with occasional gusty winds in summer season (mid-March – mid-October) scanty rainfall associated with moderately low average air temperature, relative humidity, rainfall and sunshine in winter season (mid-October – mid-March) (Table 2).

**Table 1:** Analytical soil properties of the experimental fields (0-15 cm depth).

Soil properties	Experiment # 1	Experiment # 2
pH	6.81	6.88
EC (dS m^-1^)	1.108	1.110
Org. C (%)	1.48	1.21
Total N (%)	0.14	0.13
P (ppm)	15.51	16.28
K (meq 100g^-1^)	0.13	0.11
S (ppm)	5.07	8.87
Ca (meq 100g^-1^)	2.41	2.38
Sand (%)	43	45
Silt (%)	52	50
Clay (%)	5	5
Textural class	Silty Loam	Silt Loam
Particle density (g cc^-1^)	2.69	2.68
Bulk density (g cc^-1^)	1.37	1.36

**Table 2:** Monthly average values of key weather parameters at the experimental site during the period from December 2024 to May 2025.

Months	**Air Temperature (°C)			**Humidity (%)	*Rainfall (mm)
	Maximum	Minimum	Average		
Dec-24	26.5	13.4	20	82.5	0.0
Jan-25	24.9	13.8	19.4	81.4	0.0
Feb-25	27.8	16.0	21.6	77.8	0.0
Mar-25	31.2	20.0	25.6	73.8	0.6
Apr-25	32.1	23.2	27.6	80.1	8.5
May-25	33.4	24.3	28.6	78.1	3.6

*= Daily total, ** = Daily average

Source: Weather yard, Bangladesh Agricultural University

### 2.2. Experimental design and layout

#### 2.2.1. Priming under control laboratory (Expt.#1).

This experiment was a factorial one, and the treatments comprised four foxtail millet varieties – BARI kaon-1 (V_1_), BARI kaon-2 (V_2_), BARI kaon-3 (V_3_) and BARI kaon-4 (V_4_), and six priming agents with two concentrations each, including a control (without priming) and hydropriming – No priming (P_0_), Hydropriming (P_1_) 15000 ppm KNO_3_ (P_2_), 30000 ppm KNO_3_ (P_3_), 40000 ppm Mannitol (P_4_), 60000 ppm Mannitol (P_5_), 10000 ppm NaCl (P_6_), 20000 ppm NaCl (P_7_), 100 ppm PEG (P_8_), 150 ppm PEG (P_9_), 500 ppm NaOCl (P_10_), 1000 ppm NaOCl (P_11_), 10000 ppm CaCl_2_ (P_12_) 20000 ppm CaCl_2_ (P_13_). The experiment was laid out following a completely randomized design with four replications.

#### 2.2.2. Field experiments (Expt.#2 and #3).

Both field experiments were laid out in a randomized complete block design with three replications. The treatments were the same in both experiments; the only difference was the growing season. The priming agents were selected based on laboratory experiments that showed the best performance. Both the two factorial experiments comprised four foxtail millet varieties – BARI kaon-1 (V_1_), BARI kaon-2 (V_2_), BARI kaon-3 (V_3_) and BARI kaon-4 (V_4_), and four priming treatments *viz*.; no priming – Control (T_0_), hydropriming (T_1_), 30000 ppm KNO_3_ (T_2_), 10000 ppm NaCl (T_3_).

### 2.3. Experimental materials

Four foxtail millet varieties *viz*., BARI kaon-1, BARI kaon-2, BARI kaon-3, and BARI kaon-4 released by Bangladesh Agricultural Research Institute (BARI), Gazipur, Bangladesh were used as experimental materials. The details of the foxtail millet varieties are presented in Table 3. Seeds were collected from the Regional Agricultural Research Station, BARI, Jamalpur. Seeds were visually inspected and only healthy, uniform, and disease-free seeds were selected to ensure good germination and uniform crop stand.

**Table 3:** Details of the four foxtail millet varieties.

Variety	Released year	Field duration (Days)	Grain yield (t ha^-1^)	Plant height (cm)	Grain Color
BARI Kaon 1	2005	80-90	2.0–2.5	120-130	Golden Yellow
BARI Kaon 2	2009	75-90	2.2–2.8	120-130	Light Yellow
BARI Kaon 3	2017	75-90	2.5–3.0	80-100	Brownish Yellow
BARI Kaon 4	2020	75-90	2.8–3.5	95–115	Pale Yellow

Laboratory grade priming agents were used in all the experiments. Details of the priming agents are presented in Table 4.

**Table 4:** Description of the priming agents.

Sl. No.	Priming agent	Chemical formula	Manufacturer	Origin
1	Potassium nitrate	KNO_3_	Merck	India
2	Mannitol	C_6_H_14_O_6_	Smart Lab	Indonesia
3	Sodium chloride	NaCl	Merck	India
4	Polyethylene glycol 6000	PEG 6000	Sisco Research Lab	India
5	Sodium hypochlorite	NaOCl	Merck	India
6	Calcium chloride	CaCl_2_	Research Lab Fine Chem Industries	India

### 2.4. Preparation of priming solutions

All priming solutions were prepared using analytical-grade chemicals and distilled water. Seeds and solution ratio was 1:5 (g L^-1^), which allowed for 500 seeds to be fully submerged during the soaking process. The specific amount of solute, in weight or concentration, required for each priming solution was calculated to achieve the target ppm.

### 2.5. Priming procedure

The seeds for each treatment were soaked in the priming solutions for 16 hrs at room temperature (25 ± 2 °C) in the laboratory because the germination starting time of foxtail millet seeds calculated was 32 hrs without any treatment uses, and for hydropriming seeds samples were soaked only in distilled water. After that the soaked seeds were rinsed thoroughly with distilled water for three times. The rinsed seeds were then laid on clean, sterile tissue paper and dried under air at room temperature to return to their original weight and moisture content. When seeds were dry, they were placed in zipper-sealed plastic bags with the label containing (variety name, treatment and replication number). Then the treated seeds were kept in a refrigerator at 4 ± 2°C for 15 days to stabilize the physiological status of priming, and the seeds became back to the metabolic equilibrium but didn’t lose the effects of priming [11,12]. At the end of the storage, the seeds were used for the germination and seedling growth tests. The control seed were not subjected to any priming treatment but were handled under the same conditions as primed seeds. After priming and storage, the treated seeds were subjected to germination and seedling growth evaluation in case of experiment # 1, and sown in the experimental plots in case of Experiment #2 and #3.

### 2.6. Petri dish preparation and growth medium

Sterilized sand served as the germination medium, and the seeds were placed in plastic Petri dishes (90 mm × 15 mm). Sand was moistened with distilled water before sowing and during the experiment. Regular watering was done to maintain optimum moisture condition.

### 2.7. Seed sowing and replications

From each treatment × variety combination, 400 seeds were taken from the primed 500 seeds and divided into four replications, with each replication consisting of 100 seeds. Thus, 100 seeds were placed in each Petri dish. The seeds were evenly distributed over the sand surface to ensure uniform exposure to moisture and aeration. The experiment was carried out following ISTA [13] guidelines with necessary modifications.

### 2.8. Germination monitoring

Seeds were kept at room temperature 25 ± 1°C under normal light to facilitate germination for 7 days. Seed germination was recorded up to 7 days, starting from the second day after sowing (DAS). A seed was taken as germinated when the radicle emerged out of the seed coat and the tip of the radicle was 2 mm or more in length [13]. The number of germinated seeds was recorded each day for each Petri dish, and this was used to calculate the germination percentage and the mean germination time.

### 2.9. Data recorded (Experiment # 1)

All the germinations indices were calculated as per the equations described by Islam and Kato-Noguchi [14].

#### 2.9.1. Germination percentage.

Germination percentage (GP) was calculated as the number of seeds which was germinated within 7 days as a proportion of number of seeds shown in each treatment.


$$\text{Germination Percent }(\text{GP})=\left(\frac{\text{Number of germinated seeds at final count}}{\text{Total number of seeds }}\right)\times \text{1}\text{0}\text{0}$$

#### 2.9.2. Mean germination time.

Mean germination time (MGT) was calculated according to the equation described below:


$$\text{MGT}=\frac{\sum n\times d}{\sum n}$$

where *n* = number of seeds germinated on day *d*, and *d* = number of days from the start of the test.

#### 2.9.3. Germination index.

Germination index (GI) was calculated using the following formulae:


$$\text{GI}=\left[\frac{\text{Number of germinated seeds}}{\text{Days of first count}}\right]+\ldots +\left[\frac{\text{Number of germinated seeds}}{\text{Days of final count}}\right]$$

#### 2.9.4. Seedling vigor index.

On the seventh day after seed placement for germination, five seedlings were randomly selected from each replicate. The root and shoot lengths were measured. Vigor index (VI) was calculated from total germination and seedlings length by using the formulae:


$$\text{SVI}=\left(\frac{\text{Seedling length}(\text{mm})\times \text{Germination percent}}{\text{100}}\right)$$

#### 2.9.5. Coefficient of the rate of germination.

Coefficient of the rate of germination (CRG) provides a comparative measure of germination speed:


$$\text{CRG}=\frac{\sum 𝐍}{\sum 𝐍\times 𝐃}\times \text{1}\text{0}\text{0}$$

Where, N = number of seeds germinated on day D, and D = respective days.

#### 2.9.6. Time required for 50% germination.

Time required for 50% germination calculated as per the following equation:


$${T_{\text{5}\text{0}}=t}_{i}+\frac{\left[\left\{\left(\frac{\text{N}}{\text{2}}\right)-n_{i}\right\}\left(t_{i}-t_{j}\right)\right]}{\left(n_{i}-n_{j}\right)}$$

Where, N is the final number of germination and $n_{i}$, $n_{j}$ cumulative numbers of seeds germinated by adjacent counts at times $t_{i}$ and $t_{j}$ when $n_{i}<N/\text{2}<n_{j}$

#### 2.9.7. Speed of emergence.

Calculated as per the following equation:


$$Speed\;of\;emergence\;(SE)=\sum \frac{n}{t}=\;\frac{n_{\text{1}}}{t_{\text{1}}}\;+\;\frac{n_{\text{2}}}{t_{\text{2}}}\;+\;\ldots \ldots \ldots \;+\;\frac{n_{n}}{t_{n}}$$

Where, n = number of seedlings emerged on day t, t = days from seed set for germination

#### 2.9.8. Shoot and root length.

At 7 DAS, five seedlings were sampled per Petri dishes. Shoots and roots were separated, and lengths were measured using a millimeter scale.

#### 2.9.9. Seedling dry weight.

Seedling dry weight of 20 sample from each Petri dishes were measured after drying the whole seedlings in an oven at 70 °C for 72 hrs. Finally, the seedling dry weight of each seedling were calculated and expressed in mg.

### 2.10. Crop husbandry (Experiment # 2 & 3)

The experimental filed was prepared by repeated ploughing followed by laddering to obtain a fine tilth, and all weeds and crop residues were removed. Fertilizers were applied at the recommended rates of 170, 125, 90, 55 and 4 kg ha ⁻ ¹ urea, triple super phosphate (TSP), muriate of potash (MoP), gypsum, and zinc sulphate, respectively. All fertilizers except urea were applied as basal doses during final land preparation. Urea was applied in two equal splits at 7 and 35 days after sowing (DAS), with an additional 40 kg ha^-1^ applied at 55 DAS in Exp.#2 due to reduced plant growth under low temperature. This additional urea application in the winter season was to compensate for reduced plant growth under lower temperature conditions and ensure normal growth. As treatments were compared within each season under uniform management, the potential confounding effect was minimized. In Exp.#2, seeds were sown on 17 December 2024 in rows spaced at 25 cm × 5 cm. Weed control was achieved through three manual weedings at 35, 55, and 75 DAS. Two light irrigations were applied at 45 and 90 DAS. In Exp.#3, seeds were sown on 09 March 2025 in rows, maintaining a plant density similar to Exp.#2. Weedings were carried out at 30, and 45 DAS. No additional urea or irrigation was applied in Exp.#3. No plant protection measures were required in either experiment, as no insect or disease infestation was observed. The crops were harvested on 07 April 2025 (Exp.#2) and 23 May 2025 (Exp.#3). Five hills aside from the border hill were chosen at random from each plot in order to record required data on different yield contributing characteristics. The harvested crop from each plot was then taken to the threshing floor after being individually wrapped and appropriately tagged. To determine each plot’s grain yield, the grains were cleaned and weighed. Grain yield at 14% moisture content was recorded on a plot basis after proper threshing, cleaning, and drying. The yield obtained from each plot was converted to ton per hectare (t ha ⁻ ¹) for statistical analysis. The straw yield was also recorded after separating the grains. Straw from each plot was sun-dried and weighed and was converted to t ha ⁻ ¹.

### 2.12. Statistical analysis

The collected data were compiled and tabulated. All data were subjected to two-way analysis of variance (ANOVA) through the utilization of “doebioresearch” package in “R studio” software (version 2025.09.2 + 418) to evaluate the main effects of variety and priming treatments, as well as their interaction effects. The factors included four foxtail millet varieties and fourteen seed priming treatments, resulting in 56 treatment combinations. Prior to ANOVA, the assumption of normality and homogeneity of variance were tested using the Shapiro-Wilk test. When significant differences were detected (p ≤ 0.05), mean separation was performed using the Least Significant Difference (LSD) test at the 5% level of significance. We visualized Heatmap by using “ggplot2” and “metan” package. The PCA was analyzed by “FactoMineR” and “factoextra” package and cluster analysis was by “FactoMineR”, “factoextra” and “cluster” package in R studio (version 2025.09.2 + 418).

## 3. Results

### 3.1. Effects of variety on seed germination of seed and seedling vigor

The germination percentage of foxtail millet varied significantly among varieties (Table 5). The maximum germination (%) demonstrated at BARI Kaon-1 (73.02%) followed by BARI Kaon-2 (71.09%). Similar pattern showed for germination index and germination energy, maximum germination index observed at BARI Kaon-1 (100.18) followed by BARI Kaon-2 (97.61). Whereas, germination energy of BARI Kaon-1 and BARI Kaon-2 was 53.41 and 51.70, respectively. Time required for 50% germination and mean germination time also varied significantly. BARI Kaon-3 required longest time for 50% germination and mean germination time (Table 5). Other varieties showed statistically identical results. BARI Kaon-1, BARI Kaon-2, and BARI Kaon-4 exhibited maximum and identical coefficient of rate of germination. The highest seedling vigor was exhibited highest at BARI Kaon-3 (4.88) followed by BARI Kaon-2 (4.63). The maximum speed of seedling emergence was observed at BARI Kaon-4 (77.59).

**Table 5:** Effect of variety on different seed germination indices and seedling vigor of foxtail millet.

Variety	Germination (%)	Germination index	Time required for 50% germination (days)	Mean germination time (days)	Coefficient of rate of germination	Seedling vigor index	Speed of emergence	Germination energy
BARI Kaon-1	73.02a	100.18a	1.72b	4.67b	21.40a	4.50bc	73.03b	53.41a
BARI Kaon-2	71.09ab	97.61a	1.73b	4.67b	21.39a	4.63ab	72.63b	51.70ab
BARI Kaon-3	68.93bc	91.10b	1.83a	4.71a	21.23b	4.88a	67.71c	47.07c
BARI Kaon-4	66.70c	91.75b	1.69b	4.65b	21.51a	4.32c	77.59a	50.09bc
Level of sig.	**	**	**	**	**	**	**	**
CV (%)	10.41	11.47	11.53	1.84	1.77	15.32	15.34	17.02

** = Significant at 0.01 level of probability.

The highest shoot, root and seedling length exhibited at BARI Kaon-3 (3.56, 3.44 and 7.01 cm, respectively) followed by BARI Kaon-2 for shoot and seedling length, and BARI Kaon-4 for root length. BARI Kaon-3 (42.95 mg) and BARI Kaon-4 (39.37 mg) showed highest seedling dry weight (Table 6). The maximum shoot dry weight (mg) was exhibited by BARI Kaon-3 (21.81 mg). Root dry weight exhibited non-significant difference. BARI Kaon-1 exhibited the maximum shoot and root ratio, which was statistically identical with BARI Kaon-2 and BARI Kaon-4, while BARI Kaon-3 has the lowest shoot and root ratio (Table 6).

**Table 6:** Effect of variety on seedling growth of foxtail millet.

Variety	Shoot length (cm)	Root length (cm)	Seedling length (cm)	Seedling dry weight (mg)	Shoot dry weight (mg)	Root dry weight (mg)	Shoot: root ratio
BARI Kaon-1	3.16c	2.95c	6.11c	35.83b	17.29b	18.54	1.18a
BARI Kaon-2	3.41b	3.03c	6.44b	38.33b	18.80b	19.53	1.16ab
BARI Kaon-3	3.56a	3.44a	7.01a	42.95a	21.81a	21.15	0.97b
BARI Kaon-4	3.13c	3.25b	6.38bc	39.37ab	19.03b	20.34	1.14ab
Level of sig.	**	**	**	**	**	NS	*
CV (%)	11.58	14.89	11.15	10.16	8.45	6.05	5.15

** = Significant at 0.01 level of probability; * = Significant at 0.05 level of probability; NS = non-significant

### 3.2. Effects of priming agent on seed germination and seedling vigor of foxtail millet

The effects of seed priming agent exhibited significance difference among treatments throughout all studied parameters (Table 7, 8). The highest germination (%) of foxtail millet was found in 10000 ppm NaCl (86.44%) was statistically identical with 60000 ppm Mannitol (83.19%), 20000 ppm NaCl (83.06%) and 100 ppm PEG (82.06%) (Table 7). The minimum germination (%) was observed at 20000 ppm CaCl_2_ (36.63%). The maximum index of germination was exhibited at 10000 ppm NaCl (116.49) and was at par with 20000 ppm NaCl (109.79) and 100 ppm PEG (109.90), while minimum germination index was demonstrated at 20000 ppm CaCl_2_ (47.59). Moreover, more time required for 50 percent germination was found at 30000 ppm KNO_3_ (2.00 days) followed by 2000 ppm NaCl (1.93 days), whereas minimum time required for 50% germination was at no priming (1.55 days) and this value was statistically identical with15000 ppm KNO_3_ (1.59 days), hydro-priming (1.60 days), 40000 ppm mannitol (1.67 days), 10000 ppm NaCl (1.65 days) and 20000 ppm CaCl_2_ (1.66 days) (Table 7). The longest time required for germination was at 30000 ppm KNO_3_ (4.78 days) followed by 1000 ppm NaOCl (4.76 days) and 20000 ppm CaCl_2_ (4.75 days), whereas the shortest time required for germination was no priming (4.54 days) and hydropriming (4.56 days). The maximum coefficient rate of germination was found at no priming (22.01) and hydropriming (21.91), whereas minimum was observed at 30000 ppm KNO_3_ (20.93) and was at par with 1000 ppm NaOCl (20.99) and 20000 ppm CaCl_2_ (21.06) (Table 7). The highest seedling vigor index was observed at 500 ppm NaOCl (6.08), that was statistically similar with 100 ppm PEG (5.91), 1000 ppm NaOCl (5.66), 10000 ppm CaCl_2_ (5.82), but the lowest seedling vigor index was observed at no priming (2.28) followed by 60000 ppm mannitol (2.40). The maximum speed of emergence was found at no priming (91.24) followed by hydropriming (85.71) and 500 ppm NaOCl (86.51). The germination energy was found highest at 500 ppm NaOCl (61.63) which was statistically similar with 150 ppm PEG (61.06), 10000 ppm NaCl (60.44), 15000 KNO_3_ (57.50) and 40000 ppm mannitol (56.94), while 20000 ppm CaCl_2_ was showed lowest germination energy (Table 7).

**Table 7:** Effect of priming agent on different seed germination indices and seedling vigor.

Priming agent	Germination (%)	Germination index	Time required for 50% germination (days)	Mean germination time (days)	Coefficient of rate of germination	Seedling vigor index	Speed of emergence	Germination energy
P_0_	54.75f	84.13f	1.55f	4.54i	22.01a	2.28i	91.24a	50.00de
P_1_	63.69e	96.93de	1.60ef	4.56hi	21.91ab	2.78h	85.71a	54.88b-d
P_2_	74.06d	102.10 cd	1.59ef	4.64fg	21.50 cd	4.79f	77.55b	57.50a-c
P_3_	75.19 cd	92.63e	2.00a	4.78a	20.93i	4.04g	61.90d	47.25e
P_4_	80.19bc	108.03bc	1.67d-f	4.68d-f	21.37d-f	3.69g	71.21bc	56.94a-c
P_5_	83.19ab	106.56bc	1.95ab	4.72a-d	21.17f-i	2.40hi	63.75 cd	52.75c-e
P_6_	86.44a	116.49a	1.65d-f	4.68d-f	21.37d-f	5.01d-f	70.22bc	60.44ab
P_7_	83.06ab	109.79ab	1.93ab	4.71b-e	21.23e-h	4.90ef	65.30 cd	54.06 cd
P_8_	82.06ab	109.90ab	1.71c-e	4.69c-f	21.30d-g	5.91ab	66.57 cd	54.13 cd
P_9_	80.00bc	108.49bc	1.82bc	4.69d-f	21.32d-g	5.42b-d	77.17b	61.06a
P_10_	71.88d	104.47b-d	1.72c-e	4.60gh	21.71bc	6.08a	86.51a	61.63a
P_11_	53.50f	69.48g	1.77 cd	4.76ab	20.99hi	5.66a-c	64.43 cd	34.94f
P_12_	54.44f	75.65g	1.78 cd	4.66e-g	21.47c-e	5.82a-c	76.94b	40.88f
P_13_	36.63g	47.59h	1.66d-f	4.75a-c	21.06g-i	5.37c-e	59.87d	21.50g
Level of sig.	**	**	**	**	**	**	**	**
CV (%)	10.41	11.47	11.53	1.84	1.77	15.32	15.34	17.02

Here, No priming (P_0_); Hydropriming (P_1_); 15000 ppm KNO_3_ (P_2_); 30000 ppm KNO_3_ (P_3_); 40000 ppm Mannitol (P_4_); 60000 ppm Mannitol (P_5_); 10000 ppm NaCl (P_6_); 20000 ppm NaCl (P_7_); 100 ppm PEG (P_8_); 150 ppm PEG (P_9_); 500 ppm NaOCl (P_10_); 1000 ppm NaOCl (P_11_); 10000 ppm CaCl_2_ (P_12_); 20000 ppm CaCl_2_ (P_13_); ** = Significant at 0.01 level of probability

**Table 8:** Effect of priming agent on seedling growth of foxtail millet.

Priming agent	Shoot length (cm)	Root length (cm)	Seedling length (cm)	Shoot dry weight (mg)	Root dry weight (mg)	Seedling dry weight (mg)	Shoot: root ratio
P_0_	2.18e	2.08f	4.26d	12.03f	10.66g	22.69f	1.25b-d
P_1_	2.44e	1.91f	4.35d	15.66c-f	13.9fg	29.56d-f	0.9def
P_2_	3.76a	3.01e	6.77bc	28.96a	35.20b	64.16ab	1.23 cd
P_3_	3.40bc	3.06e	6.46c	26.27a	30.72b	56.99b	1.14 cd
P_4_	3.61ab	3.74ab	7.35a	25.42a	41.61a	67.02a	1.64ab
P_5_	3.56ab	2.96e	6.52c	21.13b	21.82c	42.94c	1.04c-e
P_6_	3.52ab	3.51bc	7.03ab	16.66c-e	19.42c-e	36.07 cd	1.29bc
P_7_	3.59ab	3.23c-e	6.82bc	20.89b	14.84d-g	35.74 cd	0.74ef
P_8_	3.53ab	3.60ab	7.13ab	12.94ef	12.30fg	25.24ef	1.09c-e
P_9_	3.24 cd	3.50b-d	6.74bc	17.04 cd	16.00d-f	33.04d	1.02c-e
P_10_	3.49bc	3.17de	6.66bc	21.52b	10.79g	32.31de	0.54f
P_11_	3.59ab	3.92a	7.51a	17.87bc	14.72e-g	32.59de	0.96c-e
P_12_	3.50a-c	3.23c-e	6.73bc	19.13bc	16.73d-f	35.86 cd	1.01c-e
P_13_	3.01d	3.44b-d	6.45c	13.75d-f	19.74 cd	33.49d	1.73a
Level of sig.	**	**	**	**	**	**	**
CV (%)	11.58	14.89	11.15	8.45	6.05	10.16	5.15

Here, No priming (P_0_); Hydropriming (P_1_); 15000 ppm KNO_3_ (P_2_); 30000 ppm KNO_3_ (P_3_); 40000 ppm Mannitol (P_4_); 60000 ppm Mannitol (P_5_); 10000 ppm NaCl (P_6_); 20000 ppm NaCl (P_7_); 100 ppm PEG (P_8_); 150 ppm PEG (P_9_); 500 ppm NaOCl (P_10_); 1000 ppm NaOCl (P_11_); 10000 ppm CaCl_2_ (P_12_); 20000 ppm CaCl_2_ (P_13_); ** = Significant at 0.01 level of probability

The highest shoot length (cm) was exhibited at 15000 ppm KNO_3_ (3.76 cm) which was statistically similar with 40000 ppm mannitol (3.61 cm), 60000 ppm mannitol (3.56 cm), 10000 ppm NaCl (3.52 cm), 20000 ppm NaCl (3.59 cm), 100 ppm PEG (3.53 cm) and 10000 ppm CaCl_2_ (3.50 cm) (Table 7). In addition, the minimum shoot length was exhibited at no priming (2.18 cm) followed by 15000 ppm KNO_3_ (2.44 cm). The longest root was obtained at 1000 ppm NaOCl (3.92 cm), which was statistically identical with 40000 ppm mannitol (3.74 cm) and 100 ppm PEG (3.60 cm), whereas the shortest root-length was exhibited by hydropriming (1.91 cm) the value was at par with no priming (2.08 cm) (Table 7). The longest seedling was observed at 1000 ppm NaOCl (7.51 cm) and was statistically identical with 40000 ppm mannitol (7.35 cm), 10000 ppm NaCl (7.03 cm) and 100 ppm PEG (7.13 cm) (Table 7), whereas the shortest seedling was found at no priming (4.26 cm) closely followed by 15000 ppm KNO_3_ (4.35 cm).

The highest shoot dry weight was observed at 15000 ppm KNO_3_ (28.96 mg) and it was statistically similar with 30000 ppm KNO_3_ (26.27 mg) and 40000 ppm mannitol (25.42 mg), while minimum shoot dry weight was observed at no priming (12.03 mg) that was at par with hydropriming (15.66 mg), 100 ppm PEG (12.94 mg) and 20000 ppm CaCl_2_ (13.75 mg). The maximum root dry weight was demonstrated at 40000 ppm mannitol (41.61 mg) and minimum was observed at no priming (10.66 mg), hydropriming (13.90 mg), 500 ppm NaOCl (10.79 mg) and 1000 ppm NaOCl (14.72 mg) and they were statistically identical (Table 7). The highest seedling dry weight was exhibited at 40000 ppm mannitol (67.02 mg), while the lowest one in no priming (22.69 mg) and the value was statistically identical with 100 ppm PEG (25.24 mg) and hydropriming (29.56 mg). The maximum root and shoot ratio was found in 20000 ppm CaCl_2_ (1.71), while the minimum value was observed under hydropriming (0.9), which did not differ significantly from 20000 ppm NaCl (0.74) and 500 ppm NaOCl (0.54).

### 3.3. Interaction effects of priming agent and varieties on different seed germination indices and seedling vigor

**Fig 1:**
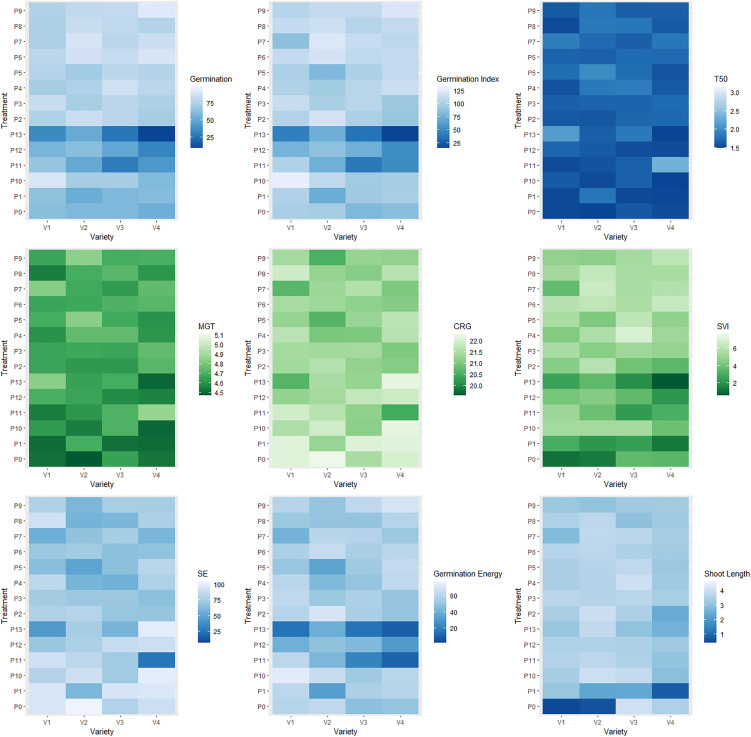
Interaction effects of foxtail millet varieties with priming agents. Here, BARI Kaon-1 (V_1_); BARI Kaon-2 (V_2_); BARI Kaon-3 (V_3_); BARI Kaon-4 (V_4_); No priming (P_0_); Hydropriming (P_1_); 15000 ppm KNO_3_ (P_2_); 30000 ppm KNO_3_ (P_3_); 40000 ppm Mannitol (P_4_); 60000 ppm Mannitol (P_5_); 10000 ppm NaCl (P_6_); 20000 ppm NaCl (P_7_); 100 ppm PEG (P_8_); 150 ppm PEG (P_9_); 500 ppm NaOCl (P_10_); 1000 ppm NaOCl (P_11_); 10000 ppm CaCl_2_ (P_12_); 20000 ppm CaCl_2_ (P_13_); ** = Significant at 0.01 level of probability.

**Fig 2:**
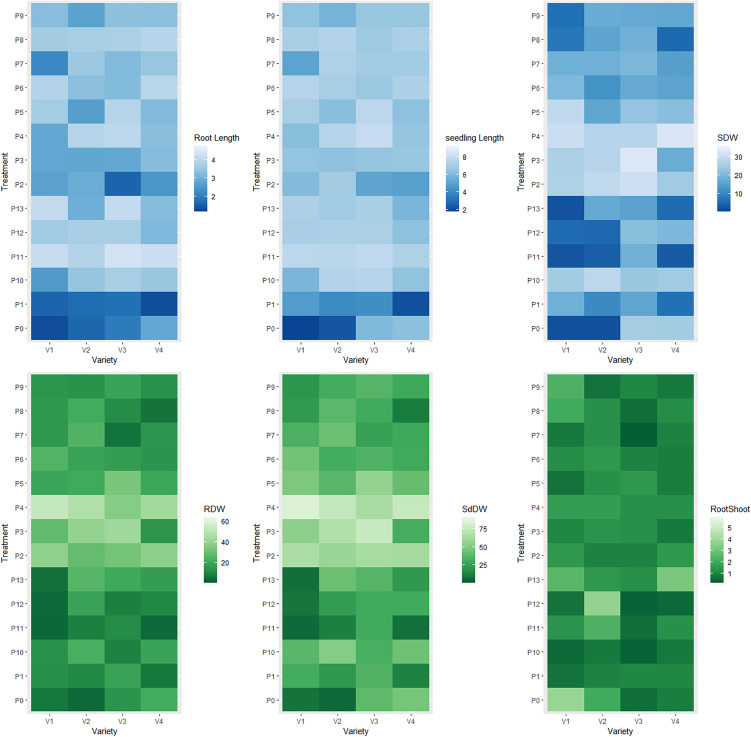
Interaction effects of foxtail millet varieties with priming agents. Here, BARI Kaon-1 (V_1_); BARI Kaon-2 (V_2_); BARI Kaon-3 (V_3_); BARI Kaon-4 (V_4_); No priming (P_0_); Hydropriming (P_1_); 15000 ppm KNO_3_ (P_2_); 30000 ppm KNO_3_ (P_3_); 40000 ppm Mannitol (P_4_); 60000 ppm Mannitol (P_5_); 10000 ppm NaCl (P_6_); 20000 ppm NaCl (P_7_); 100 ppm PEG (P_8_); 150 ppm PEG (P_9_); 500 ppm NaOCl (P_10_); 1000 ppm NaOCl (P_11_); 10000 ppm CaCl_2_ (P_12_); 20000 ppm CaCl_2_ (P_13_); ** = Significant at 0.01 level of probability.

The interaction effects of variety and priming agent exhibited significant difference among all studied traits (Figs 1 and 2). The maximum germination (%) was observed at V_1_P_10_, V_1_P_3_, V_1_P_6_, V_1_P_7_, V_2_P_5_, V_2_P_6_, V_2_P_7_, V_2_P_8_, V_3_P_2_, V_3_P_3_, V_3_P_4_, V_3_P_5_, V_4_P_5_, V_4_P_6_, V_4_P_7_, V_4_P_8_ and V_4_P_9_. The minimum germination was found at interaction effects of V_1_P_13_, V_2_P_13_, V_3_P_13_, V_3_P_11_ and V_4_P_13_. The highest germination index was observed at V_1_P_10_, V_2_P_2_, V_2_P_10_, V_2_P_7_, V_2_P_6_ and V_4_P_4_ and minimum was V_1_P_13_, V_3_P_13_, V_3_P_11_ and V_4_P_13_ interaction effects. The maximum time needed for 50% seed germination was demonstrated at V_1_P_13_, V_2_P_1_, V_3_P_13_ and V_4_P_11_. The maximum mean germination time required at V_1_P_7_, V_1_P_13_, V_2_P_9_, V_2_P_5_ and V_4_P_11_ whereas the minimum was found at V_1_P_0_, V_1_P_1_, V_1_P_8_, V_2_P_0_, V_3_P_1_, V_4_P_0_, V_4_P_1_, V_4_P_10_ and V_4_P_13_. The coefficient rate of the germination was found maximum at V_2_P_0_, V_4_P_10_, V_4_P_12_ and V_4_P_13_, while the minimum was observed at V_1_P_13_, V_1_P_7_, V_2_P_9_, V_2_P_5_ and V_4_P_11_. The maximum seed vigor index was exhibited at V_2_P_7_ and V_3_P_4_, while minimum was at V_1_P_0_, V_1_P_13_, V_2_P_0_, V_3_P_13_, V_4_P_1_ and V_4_P_13_.

The maximum speed of seed emergence was found at the interaction effects of V_1_P_8_, V_2_P_0_, V_4_P_10_, V_4_P_13_, while the minimum speed of seed emergence was observed interaction effects of V_1_P_13_, V_2_P_5_ and V_4_P_11_. The germination energy was found maximum at V_1_P_10_, V_2_P_2_, V_2_P_6_ and V_2_P_10_ while minimum was at V_1_P_13_, V_2_P_1_, V_3_P_11_, V_3_P_13_, V_4_P_11_ and V_4_P_13_. The longest shoot length was exhibited at the interaction effects of V_2_P_10_, V_2_P_2_, V_2_P_13_, V_3_P_4_ and V_2_P_6_ while the minimum was demonstrated at the interaction effects of V_1_P_0_, V_2_P_0_ and V_4_P_1_. The longest root was demonstrated at interaction effects of V_1_P_0_, V_1_P_13_, V_2_P_0_, V_3_P_13_, V_4_P_1_ and V_4_P_13_ whereas the shortest roots was found at V_1_P_0_, V_1_P_1_, V_1_P_7_, V_2_P_0_, V_3_P_2_ and V_4_P_1_. Moreover, the longest seedling was demonstrated at the interaction effect of V_1_P_11_, V_2_P_10_, V_2_P_11_, V_3_P_10_, V_3_P_11_ and V_3_P_4_ while shortest seedling was found V_1_P_0_, V_2_P_0_ and V_4_P_1_. The highest shoot dry weight was observed at the interaction effects of V_1_P_4_, V_2_P_10_, V_3_P_3_ and V_4_P_4_, while lowest shoot dry was found at V_1_P_0_, V_1_P_11_, V_1_P_13_, V_1_P_8_, V_1_P_9_, V_2_P_0_, V_2_P_11_, V_4_P_11_ and V_4_P_13_. The maximum root dry weight was demonstrated at the interaction effects of V_1_P_4_, V_1_P_2_, V_2_P_4_, V_2_P_3_, V_3_P_3_, V_4_P_4_ and V_4_P_2_ while minimum was at V_1_P_11_, V_1_P_12_, V_1_P_13_, V_2_P_0_, V_3_P_7_, V_4_P_1,_ V_4_P_8_ and V_4_P_11_. The maximum seedling dry weight was exhibited at the interaction effects of V_1_P_4_, V_1_P_2_, V_2_P_4_, V_2_P_3_, V_3_P_3_ and V_4_P_4_, whereas minimum was at V_1_P_0_, V_1_P_11_, V_1_P_12_, V_1_P_13_, V_2_P_0_, V_4_P_11_ and V_4_P_8_. The highest ratio of root and shoot was demonstrated at the interaction effects of V_1_P_0_, V_2_P_11_, V_2_P_12_ and V_4_P_13_.

### 3.4. Principal component analysis

**Fig 3:**
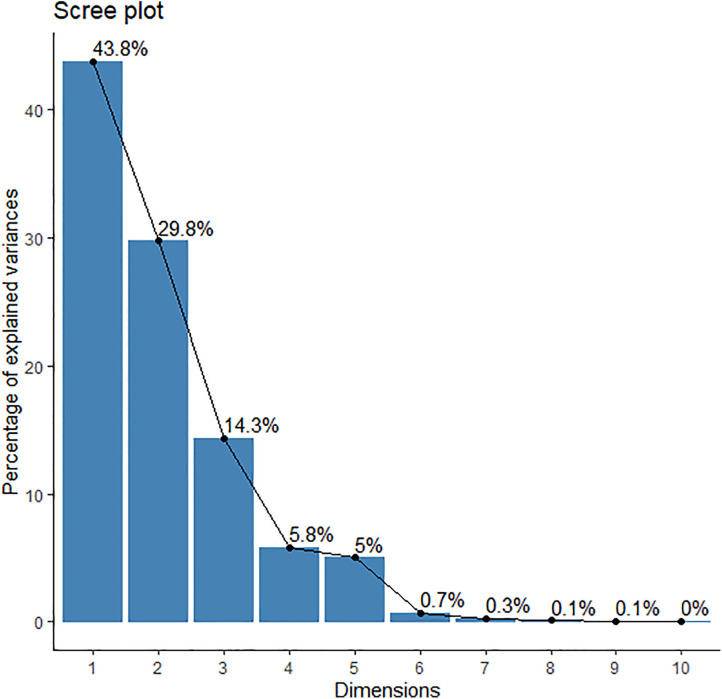
Explained variable percentage showed by scree plot.

For the analysis of principal component (PCA) explained principal component (PC) number and variances. The first PC1 explained along 43.6% variability and then gradually decreased. The 2^nd^ PC2 explained 29.8% variability and PC3 explained 14.3% of variability (Fig 3). So, first two principal components together showed 73.6% of explained variables (Fig 3). Moreover, from the graphical presentation it was exhibited that PC1 showed maximum variation, so selecting treatments and traits from PC1 are more useful.

**Fig 4:**
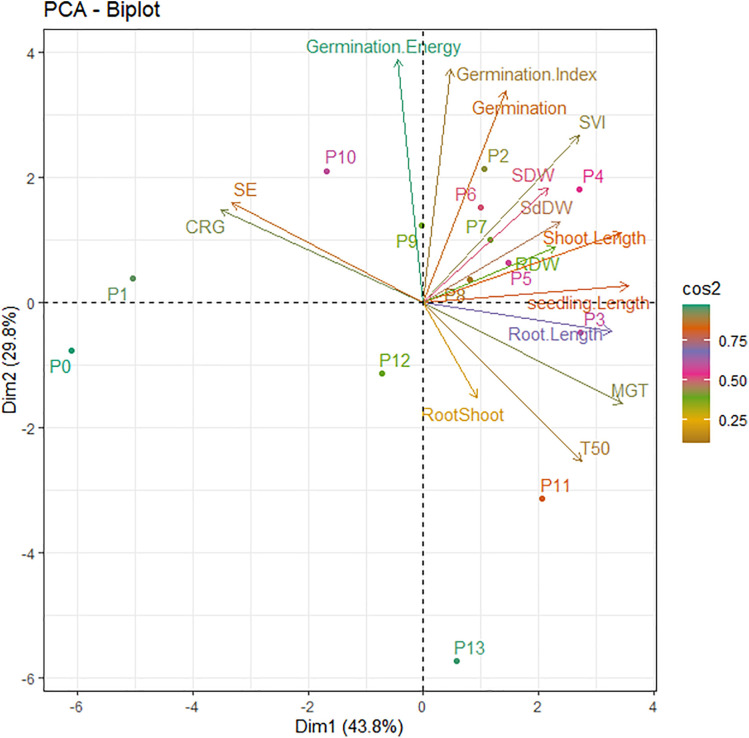
Principal component analysis for germination and growth-related traits with various priming agents.

**Fig 5:**
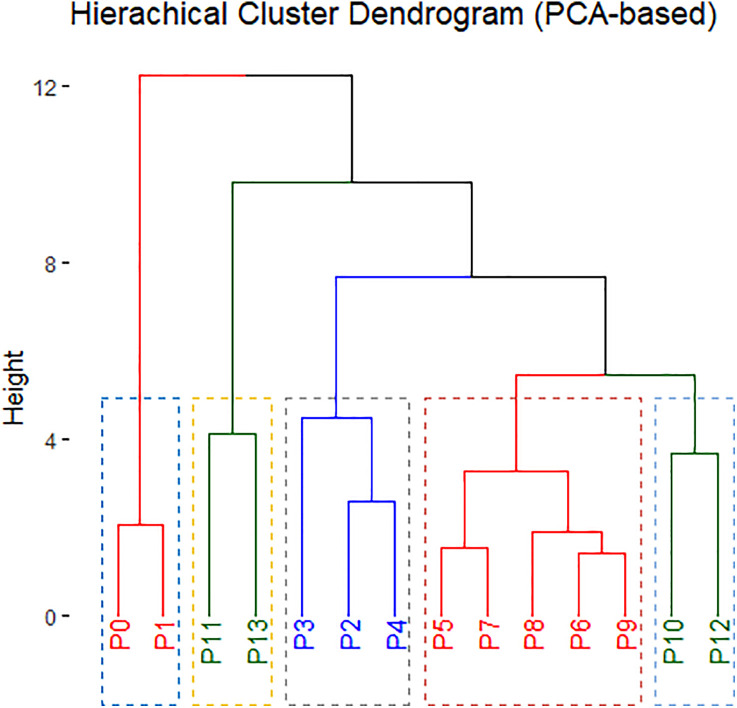
Hierarchical Cluster dendrogram (PCA-based). Here, No priming (P_0_); Hydropriming (P_1_); 15000 ppm KNO_3_ (P_2_); 30000 ppm KNO_3_ (P_3_); 40000 ppm Mannitol (P_4_); 60000 ppm Mannitol (P_5_); 10000 ppm NaCl (P_6_); 20000 ppm NaCl (P_7_); 100 ppm PEG (P_8_); 150 ppm PEG (P_9_); 500 ppm NaOCl (P_10_); 1000 ppm NaOCl (P_11_); 10000 ppm CaCl_2_ (P_12_); 20000 ppm CaCl_2_ (P_13_); ** = Significant at 0.01 level of probability.

The contribution analysis of the PCA exhibited that Dim1 was mainly driven by seedling growth related traits which includes shoot, root and seedling length, time required for 50% germination, MGT and biomass traits (shoot, root and seedling dry weight). These variables formed long vectors with high cos^2^ values. Furthermore, Dim2 was dominated by early germination traits, particularly germination (%), germination energy, germination index and seed vigor index. The PCA-biplot, revealed clear separation patterns among treatments. Treatments P_5_, P_6_, P_7_, P_8_ and P_9_ clustered near the vectors for germination vigor traits (Fig 4 and Fig 5). Treatment P_2_, P_3_ and P_4_ were exhibited toward the positive side of Dim1, correlating them with strong early seedling growth and biomass accumulation.

### 3.5. Yield attributes and yield of foxtail millet in winter season

#### 3.5.1. Effect of varieties.

The effect of variety on all yield contributing characters and yield foxtail millet except ear weight was statistically significant. The longest ear was produced by BARI kaon-2 (15.54 cm) and the shortest panicle was by BARI Kaon-4 (13.23 cm) (Table 9). The highest number of filled grain ear^-1^ was produced by BARI Kaon-2 (2753.75) which was statistically similar with BARI Kaon-3 (2657.92). The maximum 1000 grain weight was observed at BARI Kaon-2 (2.51 g) whereas the minimum was at BARI Kaon-3 (2.17 g). The highest grain yield was obtained from BARI Kaon-3 (2.35 t ha^-1^) which was statistically identical with BARI Kaon-4 (2.27 t ha^-1^), but the lowest one was obtained from BARI Kaon-1 (1.81 t ha^-1^). The highest straw yield was produced at BARI Kaon-2 (4.54 t ha^-1^) and lowest was at BARI Kaon-1 (3.39 t ha^-1^). BARI Kaon-3 (36.64%) showed maximum harvest index, while BARI Kaon-2 (32.66%) showed the minimum (Table 9).

**Table 9:** Effect of variety on yield attributes and yield of foxtail millet in winter season.

Variety	Ear length (cm)	Ear weight (g)	No. of filled grains ear^-1^	1000 grain weight(g)	Grain yield (t ha^-1^)	Straw yield (t ha^-1^)	Harvest index (%)
BARI Kaon-1	14.21b	11.32	2454.25b	2.38b	1.81c	3.39c	34.49b
BARI Kaon-2	15.54a	12.24	2753.75a	2.51a	2.20b	4.54a	32.66c
BARI Kaon-3	14.10b	11.91	2657.92a	2.17d	2.35a	4.08b	36.64a
BARI Kaon-4	13.23c	11.60	2543.75b	2.25c	2.27ab	4.12b	35.42b
Level of significance	**	NS	**	**	**	**	**
CV (%)	5.48	12.14	4.79	2.32	4.79	6.66	3,61

** = Significant at 0.01 level of probability; NS=non-significant

#### 3.5.2. Effect of priming agent.

Priming agent had significant effect on all the yield contributing characters and yield of foxtail millet. Priming agent 10000 ppm NaCl treated plot (15.90 cm) exhibited the longest ear, while no priming plot (12.83 cm) produced shortest ear which was statistically identical with hydro priming (13.39) treated plot (Table 10). The maximum ear weight was observed at 10000 ppm NaCl (13.84 g) priming plot, which was statistically similar with 30000 ppm KNO_3_ (12.87 g) treated plot (Table 4). The minimum ear weight was exhibited at no priming plot (12.83 g) and which was statistically identical with hydro priming treated plot. The number of filled grain ear^-1^, was produced highest at 10000 ppm NaCl (2864.83) treated plot and lowest was at no priming (2357.33) plot. The 1000-grain weight was found maximum at 10000 ppm NaCl (2.39 g) treated plot, while the minimum was at no priming (2.28 g) and that was at par with hydro priming (2.29 g). The highest grain yield was obtained from 10000 ppm NaCl (2.48 t ha^-1^) treated plot, whereas the minimum was from no priming plot (1.81 t ha^-1^). The highest straw yield was produced at 10000 ppm NaCl (4.25 t ha^-1^) treated plot and which was statistically identical with 30000 ppm KNO_3_ (4.11 t ha^-1^) plot and was statistically identical with hydro priming (4.12 t ha^-1^) priming plot (Table 4). The maximum harvest index was exhibited at 10000 ppm NaCl (37.09%) priming plot and minimum was at no priming (32.83%) plot, which was statistically similar with hydro priming (33.28%) plot (Table 10).

**Table 10:** Effect of priming agent on yield attributes and yield of kaon in winter season.

Priming agent	Ear length (cm)	Ear weight (g)	No. of filled grains ear^-1^	1000-grain weight (g)	Grain yield (t ha^-1^)	Straw yield (t ha^-1^)	Harvest index (%)
No priming	12.83c	9.61b	2357.33d	2.28c	1.81d	3.66b	32.83c
Hydro priming	13.39c	10.76b	2468.92c	2.29c	2.05c	4.12a	33.28c
30000 ppm KNO_3_	14.96b	12.87a	2718.58b	2.36b	2.30b	4.11a	36.02b
10000 ppm NaCl	15.90a	13.84a	2864.83a	2.39a	2.48a	4.25a	37.09a
Level of sig.	**	**	**	**	**	**	**
CV (%)	5.48	12.14	4.79	2.32	4.79	6.66	3,61

** = Significant at 0.01 level of probability.

#### 3.5.3. Interaction effect of variety and priming agent.

The interaction effect of variety and priming agent on the yield attributes and yield of foxtail millet was also statistically significant. The longest ear was produced from BARI Kaon-2 when primed with 10000 ppm NaCl (17.83 cm), while the lowest one from BARI Kaon-4 with no priming (11.77 cm). The highest ear weight was observed at BARI Kaon-2 treated with 10000 ppm NaCl (14.30 g), which was statistically identical with BARI Kaon-4 × 10000 ppm NaCl (13.77 g), BARI Kaon-1 × 10000 ppm NaCl (13.75 g), BARI Kaon-3 × 30000 ppm KNO_3_ (13.74 g), BARI Kaon-3 × 10000 ppm NaCl (13.53 g), BARI Kaon-1 × 30000 ppm KNO_3_ (13.21 g) and BARI Kaon-3 × 30000 ppm KNO_3_ (12.64 g), while the lowest one from BARI Kaon-1 × no priming (8.77 g). The highest number of filled grains ear^-1^ was produced at the interaction effects of BARI Kaon-3 × 10000 ppm NaCl (2947.33) that was at par with BARI Kaon-2 × 10000 ppm NaCl (2918.33), BARI Kaon-3 × 30000 ppm KNO_3_ (2838.67), BARI Kaon-4 and 10000 ppm NaCl (2810.00), BARI Kaon-1 × 10000 ppm NaCl (2783.67) and BARI Kaon-2 × 10000 ppm NaCl (2774.67). The lowest number of filled grains ear^-1^ was obtained from BARI Kaon-1 × no priming (2138.67) that was closely followed by BARI Kaon-1 × hydropriming (2227.00) treated plot (Table 5). The highest 1000-grain weight was found at BARI Kaon-2 × 10000 ppm NaCl (2.56 g), which was statistically similar with BARI Kaon-2 × 30000 ppm KNO_3_ (2.53 g). While the lowest one in BARI Kaon-3 × no priming (2.09 g) treated plot followed by BARI Kaon-3 × hydropriming (2.11 g) (Table 5). The maximum grain yield was obtained from BARI Kaon-2 × 10000 ppm NaCl (2.72 t ha^-1^) which was statistically similar with and BARI Kaon-4 × 10000 ppm NaCl (2.59 t ha^-1^) plot. The lowest grain yield was obtained from BARI Kaon-1 × no priming (1.17 t ha^-1^) plot. The interaction effect of BARI Kaon-2 × 10000 ppm NaCl (5.03 t ha^-1^) produced maximum straw yield closely followed by BARI Kaon-2 × hydro priming (4.63 t ha^-1^) treated plot. While the lowest straw yield was obtained from BARI Kaon-1 × no priming (2.51t ha^-1^) plot (Table 11). The maximum harvest index (%) was exhibited at the interaction effect of BARI Kaon-3 × 10000 ppm NaCl (39.04%), which was at par with BARI Kaon-4 × 10000 ppm NaCl (38.14%) and BARI Kaon-3 × 30000 ppm KNO_3_ (37.60%) treated plots (Table 11). Whereas the lowest harvest index was obtained in BARI Kaon-2 × hydropriming (30.36%) closely followed by BARI Kaon-2 × no priming (31.02%) plot.

**Table 11:** Interaction effect of variety and priming agent on the yield attributes and yield of foxtail millet in winter season.

Interaction		Ear length (cm)	Ear weight (g)	No. of filled grains ear^-1^	1000-grain weight (g)	Grain yield (t ha^-1^)	Straw yield (t ha^-1^)	Harvest index (%)
BARI Kaon-1	No priming	13.40d-g	8.77g	2138.67h	2.32ef	1.17i	2.51f	31.84fg
	Hydro priming	12.97e-h	9.57e-g	2227.00h	2.36de	1.72h	3.41e	33.61ef
	30000 ppm KNO_3_	15.23bc	13.21a-d	2667.67b-e	2.39d	2.22d-f	3.79de	36.98a-c
	10000 ppm NaCl	15.23bc	13.75ab	2783.67a-d	2.46c	2.13fg	3.86d	35.54b-e
BARI Kaon-2	No priming	13.84d-f	11.04d-g	2626.67c-f	2.46c	1.81h	4.03 cd	31.02g
	Hydro priming	14.21c-e	10.97d-g	2695.33b-e	2.48bc	2.02g	4.63ab	30.36g
	30000 ppm KNO_3_	16.27b	12.64a-d	2774.67a-d	2.53ab	2.26d-f	4.47bc	33.64ef
	10000 ppm NaCl	17.83a	14.30a	2918.33a	2.56a	2.72a	5.03a	35.63b-e
BARI Kaon-3	No priming	12.33gh	9.11fg	2342.33gh	2.09j	2.25d-f	4.23b-d	34.69de
	Hydro priming	13.52d-g	11.25c-f	2503.33e-g	2.11j	2.28d-f	4.20b-d	35.22c-e
	30000 ppm KNO_3_	14.61 cd	13.74ab	2838.67ab	2.24hi	2.38 cd	3.96d	37.60ab
	10000 ppm NaCl	15.94b	13.53a-c	2947.33a	2.26g-i	2.49bc	3.92d	39.04a
BARI Kaon-4	No priming	11.77h	9.51e-g	2321.67gh	2.22hi	2.01g	3.85de	33.78d-f
	Hydro priming	12.85f-h	11.25 cd-f	2450.00fg	2.20i	2.17e-g	4.22b-d	33.94d-f
	30000 ppm KNO_3_	13.72d-f	11.87b-e	2593.33def	2.27f-h	2.33c-e	4.22b-d	35.84b-d
	10000 ppm NaCl	14.58 cd	13.77ab	2810.00a-c	2.30e-g	2.59ab	4.20b-d	38.14a
Level of significance		*	*	*	*	*	**	*
CV (%)		5.48	12.14	4.79	2.32	4.79	6.66	3,61

** = Significant at 0.01 level of probability, * = Significant a 0.05 level of probability

### 3.6. Yield attributes and yield of foxtail millet in summer season

#### 3.6.1. Effects of variety.

BARI kaon-1 (17.33 cm), BARI Kaon-2 (17.31 cm) and BARI Kaon-3 (15.81 cm) were produced longest ear and they were statistically identical, whereas the shortest panicle was produced by BARI Kaon-4 (12.61 cm). Ear weight of foxtail millet exhibited statistically non-significant variation among varieties (Table 12). The maximum number of filled grain ear^-1^ was produced at BARI Kaon-2 (2757.33) and which was statistically identical with BARI Kaon-4 (2719.25), whereas the minimum was at BARI Kaon-3 (2563.67). The highest 1000-grain weight was demonstrated at BARI Kaon-4 (2.51 g), BARI Kaon-2 (2.44 g) and BARI Kaon-3 (2.42 g) which were statistically identical, whereas the minimum was at BARI Kaon-1 (2.22 g). The highest grain yield was produced at BARI Kaon-1 (3.43 t ha^-1^) and which was statistically varied from other varieties. Statistically the highest straw yield was produced at BARI Kaon-1 (6.50 t ha^-1^) and BARI Kaon-2 (6.19 t ha^-1^). BARI Kaon-1 (34.47%) showed maximum harvest index (Table 12).

**Table 12:** Effect of variety on yield attributes and yield of kaon in summer season.

Variety	Ear length (cm)	Ear weight (g)	No. of filled grains ear^-1^	1000-grain weight (g)	Grain yield (t ha^-1^)	Straw yield (t ha^-1^)	Harvest index (%)
BARI Kaon-1	17.33a	5.75	2610.08bc	2.22b	3.42a	6.50a	34.47a
BARI Kaon-2	17.31a	6.75	2757.33a	2.44ab	2.86b	6.19a	31.64b
BARI Kaon-3	15.81a	5.71	2563.67c	2.42ab	2.77b	5.49b	33.08ab
BARI Kaon-4	12.61b	6.73	2719.25ab	2.51a	2.66b	5.22b	33.32ab
Level of sig.	**	NS	*	*	**	**	**
CV (%)	13.38	16.77	6.19	13.95	18.06	13.87	9.34

** = Significant at 0.01 level of probability; * = Significant a 0.05 level of probability; NS = non-significant

#### 3.6.2. Effects of priming agent.

Priming agent NaCl treated plot (17.53 cm) and hydro priming (16.53 cm) exhibited the statistically identical and longest ear, whereas no priming plot (14.33 cm) produced shortest ear which was statistically identical with KNO_3_ (14.67 cm) treated plot (Table 13). The maximum ear height was observed at NaCl (7.92 g) priming plot and which was statistically varied from other priming plot (Table 13). The number of filled grain ear^-1^, was produced maximum at NaCl (2837.92) treated priming plot and which was statistically identical with KNO_3_ (2718.00) priming plot. The 1000 grain weight was found maximum at NaCl (2.86 g) treated priming plot, and which was statistically varied from others. The maximum grain yield was produced at NaCl (3.37 t ha^-1^) treated priming plot, which was statistically identical with KNO_3_ (6.04 t ha^-1^) priming plot, whereas the minimum was produced at no priming plot (2.39 t ha^-1^). The maximum straw yield was produced at NaCl (6.70 t ha^-1^) treated plot and which was statistically identical with KNO_3_ (6.04 t ha^-1^) and lowest was no priming (4.95 t ha^-1^) priming plot (Table 13). The harvest index showed non-significant variation among treatments.

**Table 13:** Effect of priming agent on yield attributes and yield of foxtail millet in summer season.

Priming agent	Ear length (cm)	Ear weight (g)	No. of filled grains ear^-1^	1000-grain weight (g)	Grain yield (t ha^-1^)	Straw yield (t ha^-1^)	Harvest index (%)
No priming	14.33b	5.24b	2561.5b	2.25b	2.39c	4.95c	32.12
Hydro priming	16.53a	5.43b	2532.92b	2.22b	2.89b	5.72b	33.53
30000 ppm KNO_3_	14.67b	6.36b	2718.00a	2.26b	3.06ab	6.04ab	33.52
10000 ppm NaCl	17.53a	7.92a	2837.92a	2.86a	3.37a	6.70a	33.34
Level of sig.	**	**	**	**	**	**	NS
CV (%)	13.38	16.77	6.19	13.95	18.06	13.87	9.34

** = Significant at 0.01 level of probability, NS=non-significance

#### 3.6.3. Interaction effects of variety and priming agent.

The longest ear was produced at interaction of BARI Kaon-2 × 10000 ppm NaCl (18.89 cm) which was statistically similar with BARI Kaon-2 × hydro priming (18.67 cm), BARI Kaon-1 × 10000 ppm NaCl (18.44 cm), BARI Kaon-1 × 30000 ppm KNO_3_ (17.89 cm); BARI Kaon-1 × hydropriming (17.78 cm); BARI Kaon-3 × 10000 ppm NaCl (17.67 cm); BARI Kaon-3 × hydro priming (16.22 cm); BARI Kaon-2 × 30000 ppm KNO_3_ (15.89 cm); BARI Kaon-2 × no priming (15.78 cm); BARI Kaon-3 × hydro priming (16.22 cm) (Table 10). The highest ear weight was observed at the interaction effects of BARI Kaon-2 × 10000 ppm NaCl (9.39 g) which was at par with BARI Kaon-4 × no priming (8.34 g), BARI Kaon-4 × 10000 ppm NaCl (8.20 g), BARI Kaon-1 × 10000 ppm NaCl (7.86 g) × BARI Kaon-3 × 30000 ppm KNO_3_. Whereas, the lowest one was observed in BARI Kaon-1 × no priming (3.65 g). The highest number of filled grains ear^-1^ was produced at the interaction effects of BARI Kaon-1 × 10000 ppm NaCl (3080.00) which was statistically identical BARI Kaon-4 × 30000 ppm KNO_3_ (2869.33), BARI Kaon-2 × 30000 ppm KNO_3_ (2867.33), BARI Kaon-2 × 10000 ppm NaCl (2823.00), whereas BARI Kaon-1 × no priming produced the lowest (2310.00). Thousand grain weight found maximum at the interaction effect of BARI Kaon-3 × 10000 ppm NaCl (2.99 g) and the value was statistically identical with BARI Kaon-2 × 10000 ppm NaCl (2.88 g), BARI Kaon-4 × 10000 ppm NaCl (2.85 g), BARI Kaon-1 × 10000 ppm NaCl (2.71 g), BARI Kaon-4 × 30000 ppm KNO_3_ (2.49 g), BARI Kaon-3 × 30000 ppm KNO_3_ (2.48 g); BARI Kaon-4 × hydro priming (2.43 g) (Table 14). While the lightest seed was produced by BARI Kaon-1 × 30000 ppm KNO_3_ (1.80 g). The highest grain yield was produced by BARI Kaon-1 and × 10000 ppm NaCl (3.93 t ha^-1^) and the value was at par with BARI Kaon-4 × 30000 ppm KNO_3_ (3.45 t ha^-1^), BARI Kaon-1 × hydro priming (3.38 t ha^-1^), BARI Kaon-1 × 30000 ppm KNO_3_ (3.31 t ha^-1^), BARI Kaon-2 × 10000 ppm NaCl (3.25 t ha^-1^), BARI Kaon-3 × 10000 ppm NaCl (3.18 t ha^-1^), and BARI Kaon-4 × 10000 ppm NaCl (3.10 t ha^-1^), BARI Kaon-1 × no priming (3.07 t ha^-1^). The lowest grain yield was obtained from BARI Kaon-4 × no priming (1.63 t ha^-1^). The interaction effect of BARI Kaon-2 with 10000 ppm NaCl produced the highest straw yield (7.35 t ha^-1^), which statistically identical with BARI Kaon-1 × 10000 ppm NaCl (7.17 t ha^-1^), BARI Kaon-1 × KNO_3_ (6.70 t ha^-1^), BARI Kaon-1 × hydro priming (6.35 t ha^-1^), BARI Kaon-3 × NaCl (6.20 t ha^-1^), BARI Kaon-4 × 10000 ppm NaCl (6.08 t ha^-1^), BARI Kaon-4 × 30000 ppm KNO_3_ (6.03 t ha^-1^), BARI Kaon-1 × no priming (5.78 t ha^-1^), while the lowest one was found in BARI Kaon-4 × no priming (4.00 t ha^-1^). The highest harvest index was obtained from BARI Kaon-4 × 30000 ppm KNO_3_ (36.62%), and the lowest was from BARI Kaon-4 × no priming (29.16%) (Table 14).

**Table 14:** Interaction effect of variety and priming agent on yield attributes and yield of foxtail millet in summer season.

Interaction		Ear length (cm)	Ear weight (g)	No. of filled grains ear^-1^	1000-grain weight (g)	Grain yield (t ha^-1^)	Straw yield (t ha^-1^)	Harvest index (%)
BARI Kaon-1	No priming	15.22b-d	3.65f	2310.00g	2.32c-f	3.07abc	5.78c-e	34.66ab
	Hydro priming	17.78a-c	6.38b-f	2392.33fg	2.03ef	3.38ab	6.35a-c	34.87ab
	30000 ppm KNO_3_	17.89a-c	5.10c-f	2658.00b-f	1.80f	3.31a-c	6.70a-c	32.90a-c
	10000 ppm NaCl	18.44a-c	7.86a-c	3080.00a	2.71a-d	3.93a	7.17ab	35.44ab
BARI Kaon-2	No priming	15.78a-d	4.88d-f	2672.33b-e	2.40b-e	2.70b-d	5.57c-e	32.78a-c
	Hydro priming	18.67ab	6.23b-f	2666.67b-f	2.21d-f	2.69b-d	5.92b-d	31.18bc
	30000 ppm KNO_3_	15.89a-d	6.51b-e	2867.33ab	2.27d-f	2.82b-d	5.93b-d	32.13a-c
	10000 ppm NaCl	18.89a	9.39a	2823.00a-c	2.88ab	3.25a-c	7.35a	30.46bc
BARI Kaon-3	No priming	15.22b-d	4.07ef	2575.67c-g	2.01ef	2.18de	4.45ef	31.88a-c
	Hydro priming	16.22a-d	4.98d-f	2543.33d-g	2.18d-f	3.05b-d	5.83b-d	34.40ab
	30000 ppm KNO_3_	14.11de	7.56a-d	2477.33e-g	2.48a-e	2.67b-d	5.48c-e	32.42a-c
	10000 ppm NaCl	17.67a-c	6.22b-f	2658.33b-f	2.99a	3.18a-c	6.20a-c	33.64a-c
BARI Kaon-4	No priming	10.78e	8.34ab	2688.00b-e	2.27d-f	1.63e	4.00f	29.16c
	Hydro priming	13.45de	4.10ef	2529.33d-g	2.43a-e	2.46c-e	4.77d-f	33.67a-c
	30000 ppm KNO_3_	11.11e	6.28b-f	2869.33ab	2.49a-e	3.45ab	6.03a-d	36.62a
	10000 ppm NaCl	15.11 cd	8.20ab	2790.33b-d	2.85a-c	3.10a-c	6.08a-d	33.84a-c
Level of significance		*	*	*	*	*	*	*
CV (%)		13.38	16.77	6.19	13.95	18.06	13.87	9.34

* = Significant at 0.05 level of probability

## 4. Discussion

### 4.1. Varietal regulation of germination dynamics and seedling vigor

Significant varietal differences in germination percentage, germination index, germination energy, seedling vigor, length of seedling and dry weight of seedling observed in the present study clearly indicated strong generic control over early seed physiological quality in foxtail millet. The superior germination performance, growth characteristics and vigor of BARI Kaon-1 and BARI Kaon-2 reflects higher metabolic readiness, efficient enzymatic activation and also rapid mobilization of stored proteins and which are fundamental for successful stand establishment and also widely reported as determinants of early crop competitiveness in millets and cereals [15,16]. Conversely, BARI Kaon-3, despite slower germination, consistently exhibited greater shoot, root and seedling length, and dry biomass accumulation. This may be due to shift in carbon allocation toward post-germinative growth rather than rapid radicle protrusion, a strategy which associated with enhanced seedling resilience under sub-optimal field conditions. Similar trade-offs between germination speed and seedling growth have been documented in millets, sorghum and maize, where slower emergence is compensated by superior root systems and improved early vigor [15,16]. The maximum seedling vigor index and biomass of BARI Kaon-3 provide a physiological explanation for maximum grain yield and harvest index (Table 5). Although, BARI Kaon-4 demonstrated faster emergence but comparatively lower seedling biomass which indicated that rapid emergence alone does not confirm maximum yield potential, supported by Mahender et al. [17].

### 4.2. Physiological basis of seed priming effects on germination and early growth of foxtail millet

Germination of foxtail millet seed and seedling traits, influenced significantly by various seed priming treatments which confirming priming as an effective physiological intervention. Various osmopriming agents likes NaCl, PEG and mannitol markedly improved germination percentage, germination index as well as germination energy. Seeds primed with salt like KNO_3_ and NaCl reflect faster germination with less time required for 50% germination and total germination, seedling vigor index, germination index and germination energy due to increase the concentration of protein in seeds, consistently α-amylase, involvement in starch mobilization and use and catalase (ROS-scavengers) to protect plasma membrane from oxidative damage [18,19]. Controlled hydration during Osmo priming involved for initiation of pre germinative metabolic process such as DNA replication, ß-tubulin; antioxidant enzyme activation such as soluble sugar mechanism, reactive oxygen species and synthesis of hydrolytic enzymes while preventing radicle emergence [20,21]. The enhancement of reserve mobilization, activity of enzyme antioxidant defense mechanism and nutrient uptake together explain the observed increase in seedling vigor biomass production and stress tolerance [22]. Moreover, priming agent NaCl (10,000–20,000 ppm) exhibited strong positive response which induction of osmotic adjustment and stress signaling pathways and responsible seed metabolic efficiency even non-saline conditions. Cotyledon cell vacuolization promotes by priming which accumulate of storage proteins, and aquaporin gene expression pattern alters responsible for enhancement of overall performance of seeds including expression of stress related proteins [19]. Moreover, osmo-primed seeds boosted seedling dry weight, chlorophyll content, amylose activity, concentration of leaf calcium and total soluble sugars, which responsible for development of seedling and production of biomass [23]. Low level of salt priming has been shown to induce a form of “stress imprinting” improving membrane stability and enzymatic activity during germination [23]. Similarly, PEG-mediated osmotic priming enhanced seedling length and vigor by improving cell elongation and development of root system, these traits are crucial for early nutrition and water acquisition. In contrast, CaCl_2_ another salt reduced germination and vigor severely, which indicated the osmotic and ionic toxicity effects due to disruption of integrity membrane and enzyme function by excessive calcium ions resulting delayed or inhibited germination [20,23]. Moreover, NaOCl (500–1000 ppm) priming agent significantly improved seedling vigor index and germination energy, likely due to seed surface sterilization, reduced pathogen and enhanced oxygen diffusion. Enhanced shoot and root elongation under NaOCl treatment further supports its role in improving early seedling health [24]. So, appropriate and optimizing priming concentration can play a significant role for enhancement of seed germination and related traits.

### 4.3. Interaction effects of variety and priming agent in response to germination dynamics and growth

The significant interaction between foxtail millet varieties and priming agents across all germination and seedling traits demonstrates that priming efficiency depends on genotype. Particularly BARI Kaon-2 × NaCl, BARI Kaon-3 × PEG and BARI Kaon-3 × NaOCl consistently produced superior germination, seedling vigor and biomass. Such type of genotype specific responses has been widely reported in cereals, where variation in seed coat permeability, reserve composition and hormonal sensitivity determines the efficacy of priming agents [15,20]. Moreover, the inferior performance of CaCl_2_ priming consistently across foxtail millet varieties further confirms that not all priming agents are universally suitable.

### 4.4. Multivariate interpretation of seed germination and seedling traits

Furthermore, the principal component analysis (PCA) provided strong multivariate confirmation of priming effects. The first two principal components (PC) together explained 73.6% of total variability. Similar findings also reported by [25,26]. PC1 was strongly correlated with seedling growth and biomass traits (shoot dry weight, root dry weight and seedling dry weight), whereas PC2 was dominated by early growth parameters (shoot length, root length, seedling length, time required for 50% germination, MGT) of foxtail millet. These findings, clearly indicated that germination, seedling vigor and seedling growth traits were representing distinct physiological process where each parameter contributes differently to crop establishment [25,27]. The clustering of KNO_3_, NaCl, PEG and NaOCl treatment confirms their role in accelerating emergence, while mannitol and CaCl_2_ related with biomass related traits.

### 4.5. Translation of early vigor into field performance during winter and summer season

The field evaluation of four foxtail millet varieties during winter season revealed that varietal differences in early vigor translated into significant variation in plant growth as well as yield components. BARI Kaon-2 demonstrated superior ear length, filled grain, 1000-grain weight, straw weight, biological yield, which reflects strong vegetative growth as well as assimilate production in both seasons. Furthermore, BARI Kaon-3 achieved the maximum grain in winter season, yield due to maximum filled grain ear^-1^ and harvest index, which indicates superior assimilate partitioning toward reproductive sinks. Higher ear length with superior filled grain is responsible for maximum grain yield of a variety [26,28]. Moreover, these decoupling of vegetative biomass and grain yield production underscores the magnitude of sink efficiency rather than absolute biomass production [29].

Furthermore, varietal performance differed between seasons, with BARI Kaon-1 performing superior in summer season may be due to maximum adaptation to higher moisture and temperature regimes. So, environment as well as variety itself plays a crucial role for better yield performance, that’s why variety should recommended based on growing seasons.

### 4.6. Yield robustness of seed priming response under winter and summer season

Enhanced early vigor likely outputted in improved canopy development, higher photosynthetic efficiency and largest nutrient uptake which ultimately increased yield [21,23]. NaCl priming outperformed significantly with panicle length, filled grain number, thousand grain weight, grain yield and harvest index in winter season and ear weight and thousand grain weight during summer season. This suggested the induction of adaptive physiological responses, such as osmotic regulation and enhanced enzyme activity [20,21]. Improvement of yield following salt priming like NaCl have been found effective in wheat, rice, maize and millets [15,30,17,23]. Furthermore, no priming and hydropriming produced inferior yields compared with salt priming, which suggesting that chemical priming (salt) is more effective than simple hydro-priming for maximizing crop productivity.

## 5. Conclusion

The present study confirms that seed priming is an effective approach to enhance germination, seedling vigor, and yield performance of foxtail millet under the agro-climatic conditions of Bangladesh. Among the evaluated treatments, NaCl priming at 10000 ppm consistently improved germination dynamics, early seedling growth, and yield attributes, resulting in superior grain yield in both winter and summer seasons. Varietal responses differed across seasons, with BARI Kaon-2 performing best during the winter season and BARI Kaon-1 exhibiting better adaptation and yield during the summer season, highlighting the importance of genotype × environment × management interactions. The strong association between early seedling vigor and final yield underscores the role of improved crop establishment in achieving yield stability. In contrast, non-primed and hydro-primed seeds consistently exhibited inferior performance, underscoring the advantage of chemical priming over simple hydration. NaCl seed priming at 10000 ppm emerges as a farmer-friendly, and season-resilient technology for enhancing seed quality, crop establishment, and yield of foxtail millet. Adoption of this technique, combined with season-specific varietal selection, can contribute to improved productivity and sustainability of millet-based cropping systems in Bangladesh and similar agro-ecological regions. This study is limited by its restriction to a single location and growing season, potentially constraining the extrapolation of findings across heterogenous agro-ecological environments. Additionally, the evaluation of a limited range of priming agents and concentrations underscores the necessity for multi-environment, multi-season investigations incorporating broader treatment diversity to robustly validate and generalize the outcomes. Furthermore, the future research should focus on elucidating the molecular and physiological mechanisms underlying NaCl-induced priming responses, particularly stress memory and osmotic regulation pathways. Integrating seed priming with nutrient management, biopriming agents, and climate-smart agronomic practices may further enhance the productivity and resilience of foxtail millet in low-input and marginal environments.
